# News Items

**Published:** 1872-02-01

**Authors:** 


### News Items.

### AMERICAN MEDICAL ASSOCIATION.

Office of Permanent Secretary,
Wm. B. Atkinson, M. D.,
1400 Pine St., cor. Broad, Philadelphia.

The Twenty-third Annual Session will be held in Philadelphia, Pa., May 7, 1872, at 11 A. M. The following committees are expected to report:

On Cultivation of the Cinchona Tree.—Dr. Lemuel J. Deal, Pennsylvania, Chairman. On the Anatomy and Diseases of the Retina —Dr. R. F. Michel, Alabama, Chairman. On the Comparative Pathology and the Effects which Diseases of Inferior Animals have upon the Human System—Dr. Geo. Sutton, Indiana, Chairman. On the Structure of the White Blood Corpuscles—Dr. J. G. Richardson, Pa., Chairman. On Vaccination—Dr. T. N. Wise, Kentucky, Chairman. On Skin Transplantation—Dr. J. Ford Thompson, D. C., Chairman. On the Nature and Process of the Restoration of Bone—Dr. A. L. McArthur, Illinois, Chairman. On some Diseases peculiar to Colorado—Dr. John Elsner, Colorado, Chairman. On Correspondence with State Medical Societies—Dr. N. S. Davis, Illinois,

Chairman. On National Health Council—Dr. Thomas M. Logan, California, Chairman. On Nomenclature of Diseases—Dr. Francis Gurney Smith, Pa., Chairman. On What, if any, Legislative means are expedient and advisable to prevent the spread of Contagious Diseases—Dr. M. H. Henry, New York, Chairman. On American Medical Necrology—Dr. J. D. Jackson, Kentucky, Chairman. On Medical Education—Dr. J. S. Weatherly, Alabama, Chairman. On Medical Literature—Dr. Theoph. Parvin, Indiana, Chairman. On Prize Essays—Dr. Alfred Stille, Pa., Chairman. On the Climatology and Epidemics of—New Hampshire, Dr. G. R. Crosby; Vermont, Dr. G. B. Bullard; Massachusetts, Dr. E. Cutter; Rhode Island, Dr. Edw. T. Caswell; Connecticut, Dr. J. C. Jackson; New York, Dr. W. F. Thoms; New Jersey, Dr. E. M. Hunt; Pennsylvania, Dr. W. L. Wells; Maryland, Dr. C. H. Ohr; Georgia, Dr. A. J. Semmes; Missouri, Dr. W. S. Edgar; Alabama, Dr. R. F. Michel; Texas, Dr. S. M. Welsh; Illinois, Dr. David Prince; Indiana, Dr. Dugan Clark; District of Columbia, Dr. J. W. H. Lovejoy; Iowa, Dr. J. Williamson; Michigan, Dr. S. H. Douglas; Ohio, Dr. J. A. Murphy; California, Dr. F. W. Hatch; Tennessee, Dr. W. K. Bowling; West Virginia, Dr. E. A. Hildreth; Minnesota, Dr. Chas. N. Hewitt; Virginia, Dr. A. G. Wortham. On the Climatology and Epidemics of—Delaware, Dr. L. B. Bush; Kansas, Dr. Tiffin Sinks; Mississippi, Dr. J. P. Moore; Louisiana, Dr. S. M. Bemiss; Wisconsin, Dr. J. K. Bartlett; Kentucky, Dr. L. P. Yandell, Sr.; Colorado, Dr. R. G. Buckingham; Oregon, Dr. E. R. Fiske; North Carolina, Dr. J. F. Haywood; South Carolina, Dr. M. Simmons.

Physicians desiring to present papers before the Association should observe the following rule:—

"Papers appropriate to the several sections, in order to secure consideration and action, must be sent to the Secretary of the appropriate section at least one month before the meeting which is to act upon them. It shall be the duty of the Secretary to whom such papers are sent, to examine them with care, and, with the advice of the Chairman of his Section, to determine the time and order of their presentation, and give due notice of the same. . . . "

OFFICERS OF SECTIONS.

Chemistry and Materia Medica—Drs. R. E. Rogers, Philadelphia, Pa., Chairman; Ephraim Cutter, Boston, Mass., Sec'y. Practice of Medicine and Obstetrics—Drs. D. A. O'-Donnell, Baltimore, Md., Chairman; Benj. F. Dawson, New York, N. Y., Sec'y. Surgery and Anatomy—Drs. John T. Hodgen, St. Louis, Mo., Chairman; W. F. Peck, Davenport, Iowa, Sec'y. Medical Jurisprudence, Hygiene and Physiology—Drs. S. C. Busey, Washington, D. C., Chairman; E. L. Howard, Baltimore, Md., Sec'y. Psychology—Drs. Isaac Ray, Philadelphia, Pa., Chairman; John Curwen, Harrisburg, Pa., Sec'y.

Secretaries of all medical organizations are requested to forward lists of their Delegates, as soon as elected, to the Permanent Secretary.

Railroad and Hotel arrangements will be announced at an early date.

W. B. ATKINSON.

James Fisk, Jr's brain is said to have weighed 59 ounces. This is the fifth heaviest on record. Cuvier's, notably the heaviest, weighed 64 ounces.

The Small-pox Epidemic in Vienna is rapidly increasing. A new ward has had to be constructed for small-pox patients in Prof. Hebra's department.

Dr. Bamberger has been elected to succeed the late Prof. Oppolzer in the Chair of Medicine, at the University of Vienna.

The Italian Profession is raising a subscription for a medal in honor of Prof. Virchow.

Diphtheria and the exanthemata are prevalent in Vienna and some other parts of Austria. Hamburg is suffering from an epidemic of small-pox.

M. Barth has been elected President, for 1872, of the Academy of Medicine, of Paris.

There are 550 students in attendance upon the lectures of the Pennsylvania Hospital.

Sixteen Japanese students have matriculated this year in Berlin.

M. Paul Dubois, Professor of Midwifery in Paris, and one of the most eminent obstetric authorities in France, died on Nov. 29, 1871, at the advanced age of 76. He held his professorship for 25 years.

A College for Siberia.—A quarter of a million dollars has been placed in the hands of Minister Tolstri to form a University in Siberia.

